# A changing landscape: Tracking and analysis of the international HDV epidemiology 1999–2020

**DOI:** 10.1371/journal.pgph.0000790

**Published:** 2023-04-25

**Authors:** Braden S. Fallon, Elaine M. Cooke, Matthew C. Hesterman, Jared S. Norseth, Sherzod B. Akhundjanov, Melodie L. Weller

**Affiliations:** 1 School of Dentistry, University of Utah, Salt Lake City, UT, United States of America; 2 Department of Applied Economics, Utah State University, Logan, UT, United States of America; 3 Department of Pathology, Division of Microbiology and Immunology, University of Utah, Salt Lake City, UT, United States of America; Chinese Academy of Medical Sciences and Peking Union Medical College, CHINA

## Abstract

The international epidemiology of Hepatitis Delta Virus (HDV) is challenging to accurately estimate due to limited active surveillance for this rare infectious disease. Prior HDV epidemiological studies have relied on meta-analysis of aggregated and static datasets. These limitations restrict the capacity to actively detect low-level and/or geographically dispersed changes in the incidence of HDV diagnoses. This study was designed to provide a resource to track and analyze the international HDV epidemiology. Datasets analyzed collectively consisted of >700,000 HBV and >9,000 HDV reported cases ranging between 1999–2020. Datasets mined from government publications were identified for Argentina, Australia, Austria, Brazil, Bulgaria, Canada, Finland, Germany, Macao, Netherlands, New Zealand, Norway, Sweden, Taiwan, Thailand, United Kingdom, and United States. Time series analyses, including Mann-Kendall (MK) trend test, Bayesian Information Criterion (BIC), and hierarchal clustering, were performed to characterize trends in the HDV timelines. An aggregated prevalence of 2,560 HDV/HBV_100,000_ cases (95% CI 180–4940) or 2.56% HDV/HBV cases was identified, ranging from 0.26% in Canada to 20% in the United States. Structural breaks in the timeline of HDV incidence were identified in 2002, 2012, and 2017, with a significant increase occurring between 2013–2017. Significant increasing trends in reported HDV and HBV cases were observed in 47% and 24% of datasets, respectively. Analyses of the HDV incidence timeline identified four distinct temporal clusters, including Cluster I (Macao, Taiwan), Cluster II (Argentina, Brazil, Germany, Thailand), Cluster III (Bulgaria, Netherlands, New Zealand, United Kingdom, United States) and Cluster IV (Australia, Austria, Canada, Finland, Norway, Sweden). Tracking of HDV and HBV cases on an international scale is essential in defining the global impact of viral hepatitis. Significant disruptions of HDV and HBV epidemiology have been identified. Increased surveillance of HDV is warranted to further define the etiology of the recent breakpoints in the international HDV incidence.

## Introduction

Worldwide, it is estimated that 12–72 million individuals are infected with Hepatitis Delta Virus (HDV) [1–4]. Regionally, the prevalence of HDV in Hepatitis B Virus (HBV) positive populations can range from less than 1% in North America and Europe to greater than 20% in parts of South America, Asia, and Africa. The accuracy of these prior estimates is heavily influenced by the limited active surveillance of HDV cases [5–7]. Recent reports have alluded to a provocative shift in the Kolmioviridae family, including the identification of a new at-risk population and HDV-like sequences in diverse animal species [8–17]. Improved infectious disease reporting networks are essential to track critical changes in HDV epidemiology, including the emergence of new HDV variants, alternative reservoirs, and novel transmission patterns of this rare infectious disease.

Since the discovery of HDV in the late 1970s, the molecular characteristics of this unique pathogen have been extensively studied [17]. HDV is a satellite RNA (sat-RNA) that requires a helper virus, classically defined as HBV, for packaging and transmission [18]. As one of the smallest viruses known to infect humans, HDV contains a ~1700nt single-stranded, circular RNA genome that supports expression of two antigens from a single open reading frame. The HDV sat-RNA and two antigens comprise the ribonucleoprotein complex that is packaged into a helper virus particle. The cellular tropism of HDV is dictated by the helper virus. Once intracellular, HDV utilizes the host cellular machinery for replication and can persist chronically in the absence of an active helper virus co-infection [18–20].

The limited active surveillance of HDV impedes the ability to accurately define the international HDV epidemiology. To address this, a study was designed to identify publicly accessible international infectious disease datasets to enable collective tracking and analyses of HDV incidence. To date, 17 international infectious disease datasets containing yearly incidence of newly reported HDV and HBV cases have been identified. Collectively, the identified dataset contains >700,000 HBV cases and >9,000 HDV cases ranging between 1999–2020. Analyses of these datasets have identified significant structural breaks in the newly reported HDV case timelines, including four distinct temporal clusters that are suggestive of significant changes in HDV epidemiology.

## Methods

### International HDV public datasets and analyses

International infectious disease datasets containing yearly newly reported cases for both HDV and HBV were obtained through data mining techniques. These data mining techniques included searching for governmental infectious disease datasets and publications through localized, language-specific, and geographically distinct search engines. All identified infectious disease data sources are noted in S1 Table. Publicly accessible infectious disease datasets for 16 countries and one administrative region, containing both yearly incidence of HDV and HBV, include Argentina, Australia, Austria, Brazil, Bulgaria, Canada (BC), Finland, Germany, Netherlands, New Zealand, Norway, Sweden, Taiwan, Thailand, United Kingdom, United States, and Macao. Datasets contain the number of HDV and HBV cases that were reported per year. Limited information is available on the number of tests performed, type of HDV and HBV testing used, patient-matched HBV and HDV testing results, or other local regulations impacting the reporting of HDV and HBV cases. Additionally, HDV cases or diagnoses reported by the health administration in each country or administrative region may represent detection of previous HDV-exposure and/or active HDV-viremia. Current HDV diagnostic paradigms restrict testing for HDV to patients with documented acute or chronic HBV infections.

Prevalence of HDV for datasets was calculated as the percentage of total newly reported HDV cases per total HBV cases or total newly reported HDV cases per 100,000 HBV cases (HDV/HBV_100,000_). Yearly HDV incidences were calculated as the ratio of newly reported yearly HDV cases reported to the total yearly HBV cases reported from each dataset and delivered as the average of yearly HDV/HBV_100,000_ with 95% confidence interval (CI). Mann-Kendall Trend analyses were used to identify increasing or decreasing temporal trends in HBV and HDV cases over the analyzed time series [21, 22]. Pairwise correlations of yearly HDV/HBV incidences for each country relative to yearly incidence of all other countries were calculated using an unconditional Pearson correlation coefficient. Correlative heatmap with hierarchal clustering and a temporal heatmap of maximal HDV/HBV yearly incidence were produced using Morpheus (Broad Institute, https://software.broadinstitute.org/morpheus/).

### Structural break analysis

To identify structural shifts in the HDV/HBV time series, a structural break analysis was performed as detailed by Bai and Perron [23, 24] and as implemented by Zeileis et al [24]. This methodology has been previously utilized to evaluate datasets for structural breakpoints in infectious disease time series [25–27]. The advantage of the Bai and Perron method is that it allows for endogenous identification of single or multiple breakpoints in the dataset timeline. An autoregressive integrated moving average (ARIMA) was performed on the aggregated dataset. Breakpoints in the mean of HDV/HBV series were determined by fitting a constant model to the data and analyzing structural changes [24, 25, 28, 29]. The null hypothesis model of no structural break was tested against a set of alternative hypothesis models of 1, 2, or more structural breaks. The Bayesian Information Criterion (BIC) was used to determine the best-fitting model for breakpoint analysis. Using data from the correlation matrix, four temporal clusters were identified. Structural break analyses were performed on both the aggregated dataset and temporal cluster datasets identified through correlative hierarchal clustering as previously detailed.

### Statistical analysis

Statistical analyses, including Student’s t-test and ANOVA, were performed on all available HDV/HBV incidence for the identified structural breaks in the HDV/HBV timelines and subsequent cluster data. The Mann-Kendall Trend Test was utilized to identify statistically significant trends in HDV and HBV incidence time series [22, 30].

## Results

### International HDV and HBV prevalence

Seventeen international, publicly accessible infectious disease datasets containing yearly newly reported HDV and HBV cases were identified and utilized in this study. Infectious disease datasets were identified for the following countries: Argentina, Australia, Austria, Brazil, Bulgaria, Canada, Finland, Germany, Macao, Netherlands, New Zealand, Norway, Sweden, Thailand, Taiwan, United Kingdom, and United States. The source data for each dataset are contained in S1 Table. Analyses were conducted on complete and partial datasets ranging between 1999–2020 (Table 1). Together, the aggregated dataset contains >700,000 HBV and >9,000 HDV cases that span five continents and represents one of the largest actively updating and publicly accessible datasets analyzed.

**Table 1:** Prevalence of Hepatitis Delta Virus (HDV) for infectious disease datasets utilized in study. Publicly accessible epidemiological datasets from 17 countries or region were identified that contained the yearly incidence of newly reported HDV and HBV cases.

	Countries or Region	Years in Dataset	HDV/HBV_100,000_	Prevalence HDV/HBV
Europe	Austria	1999–2019	546	0.55%
	Bulgaria	2008–2019	1,398	1.40%
	Finland	1999–2020	939	0.94%
	Germany	2001–2020	1,196	1.20%
	Netherlands	2004–2020	936	0.94%
	Norway	2002–2018	2,471	2.47%
	Sweden	1999–2004, 2008–2020	2,780	2.78%
	United Kingdom	2007–2019	2,880	2.88%
North America	United States	1999–2018	20,000	20.00%
	Canada (BC)	2003–2018	257	0.26%
Australia	Australia	1999–2020	773	0.77%
	New Zealand	2002, 2006, 2008–2018	4,537	4.54%
South America	Argentina	2007–2017	266	0.27%
	Brazil	2001–2018	1,286	1.29%
Asia	Macao	2000–2020	690	0.69%
	Taiwan	1999–2020	1,224	1.22%
	Thailand	1999–2020	1,337	1.34%

The prevalence of reported HDV cases relative to HBV cases was calculated for all 17 datasets for the publicly available time frames identified (Table 1). Prior studies have reported the prevalence of HDV in HBV-positive patient populations to be approximately 5%, with areas of lower or higher prevalence [1]. From our analysis, the average prevalence of HDV across the identified infectious disease datasets is 2,560 HDV/HBV_100,000_ cases (95% CI 180–4940) or 2.56% HDV cases/HBV cases (95% CI 0.18%-4.94%). Percent prevalence of HDV cases relative to HBV cases ranged from 0.26% in Canada to 20% in the United States (1999–2018, NHANES).

### Timelines of HDV and HBV Incidence

**Fig 1:**
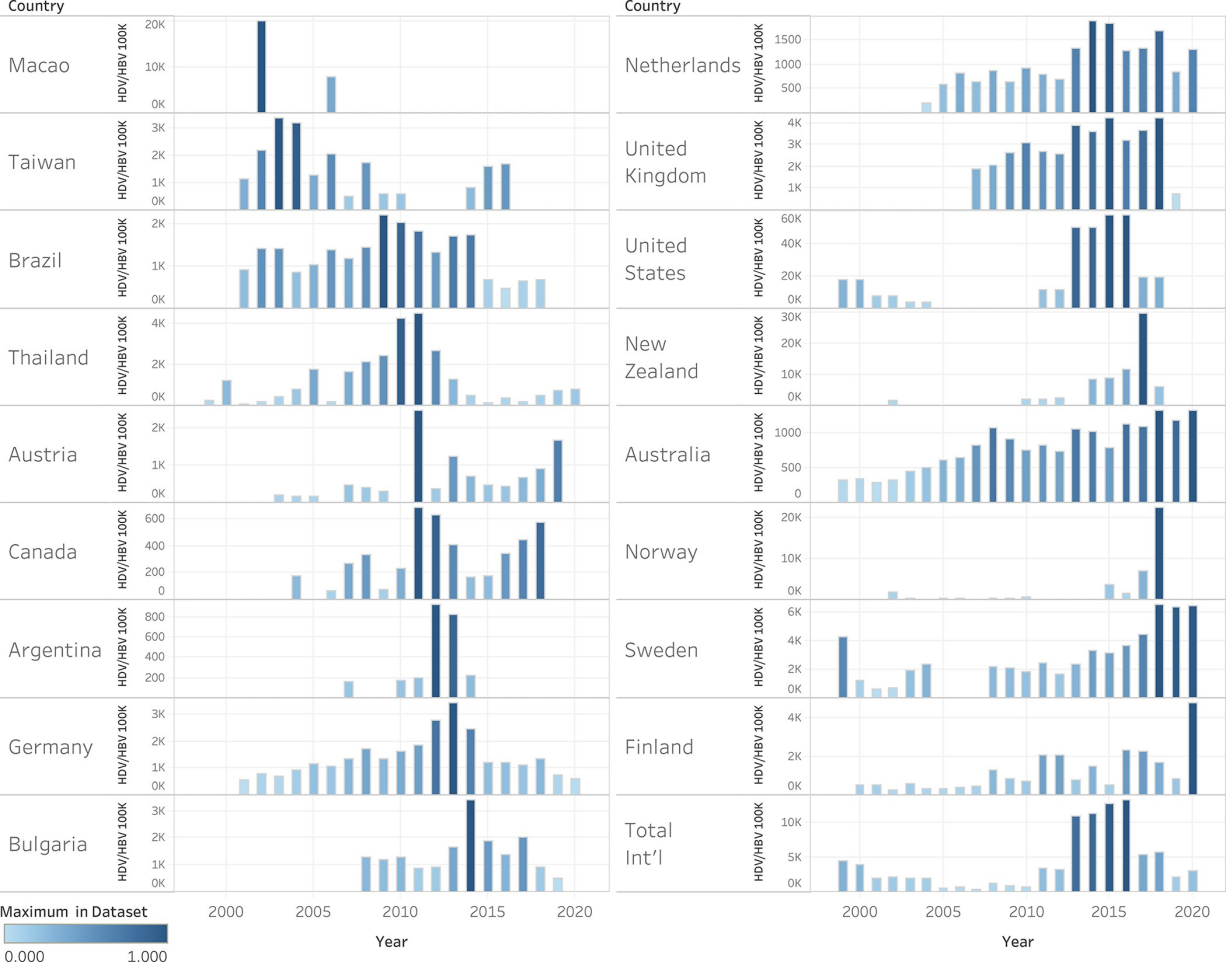
Yearly incidence of HDV/HBV_100,000_ newly reported cases. Publicly accessible infectious disease datasets containing yearly incidence of HDV and HBV for 17 countries or region spanning 5 continents were utilized in analyses. Data ranges in full or in part between 1999–2020. Order of countries is based on year of maximal HDV/HBV_100,000_ within each dataset. Total International (Int’l) is the average HDV/HBV_100,000_ across all datasets analyzed. Data sources are available in S1 Table.

Yearly reported HDV/HBV_100,000_ cases were calculated for each of the 17 datasets (Fig 1). Yearly newly reported HDV and HBV cases for each dataset are detailed in S1 Fig, and sources for datasets are noted in S1 Table. Based on the accepted dependence of HDV on an HBV co-infection, we would hypothesize that a similar trend between HDV and HBV cases would be observed. Unexpectedly, disruptions in the timelines of newly reported HDV cases independent of similar trends in HBV cases were observed (Fig 1, S1 Fig) and warranted further evaluation of the HDV and HBV time series.

### Increasing newly reported HDV cases relative to HBV cases

**Fig 2:**
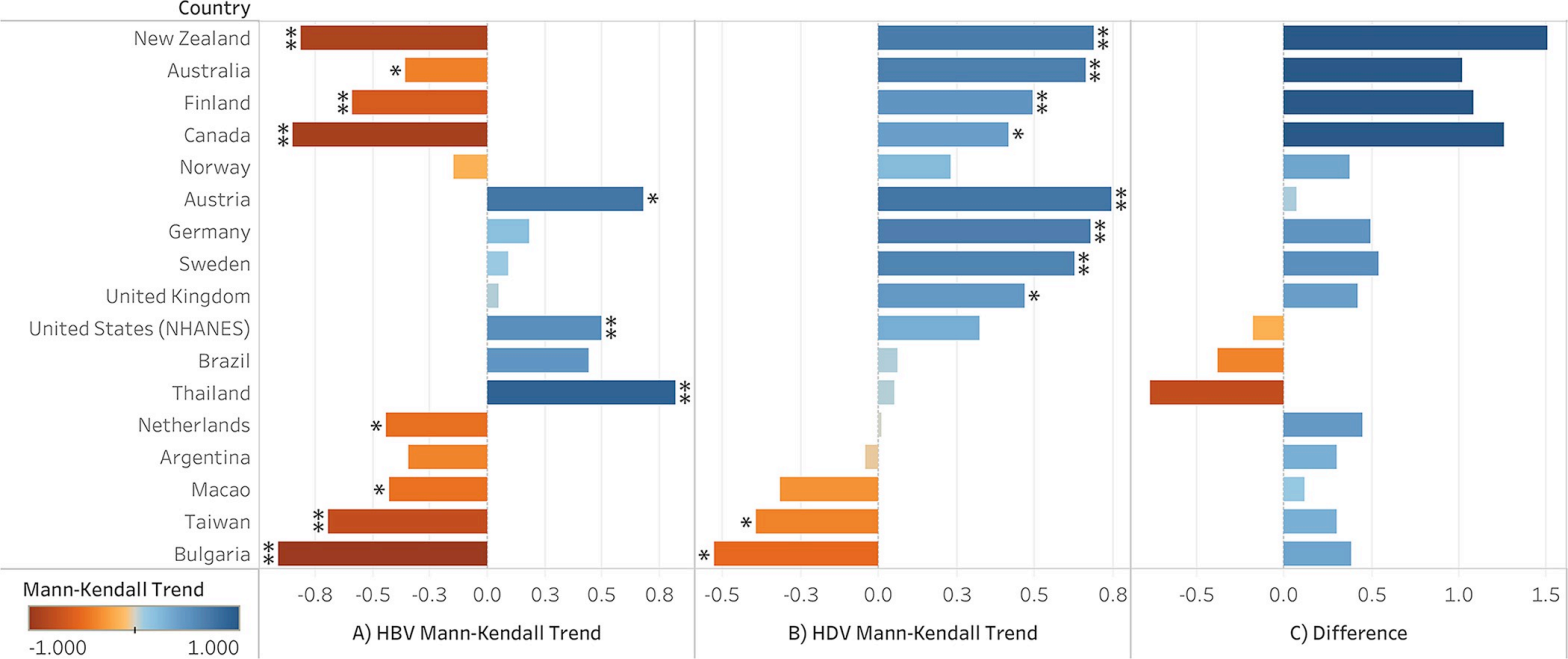
Significant divergence in HDV and HBV time series trends between 1999–2020. **A)** 47% of HBV datasets have a significant negative Mann-Kendall (MK) trend suggesting a decrease in HBV incidence over time series analyzed. 24% of HBV datasets have a significant positive MK trend suggesting an increase in HBV incidence over time series analyzed. **B)** 47% of HDV datasets have a significant positive and 12% a significant negative MK trend. The MK represents a significantly different profile from HBV (fisher exact, p<0.05). **C)** HDV MK trends were increased relative to the HBV MK trends for all but 3 (18%) including United States, Brazil and Thailand. Data are detailed in S2 Table. *p<0.05, **p<0.005.

Mann-Kendall (MK) trend analysis was performed to characterize increasing or decreasing temporal trends independently for HDV nd HBV over the time series analyzed (Fig 2, S2 Table, S2 Fig). The MK trend analysis identified divergent trends in HDV and HBV cases for a subset of datasets analyzed. For HBV, 8 of the 17 (47%) datasets rendered a significant decreasing trend and 4 of the 17 (24%) datasets rendered a significant increasing trend in HBV cases (Fig 2A). Significant MK trends were not observed in 5 (29%) HBV datasets.

In contrast, for HDV, 8 of the 17 (47%) datasets analyzed rendered a significantly increasing trend in newly reported HDV cases and only 2 of the 17 (12%) datasets rendered a significantly decreasing trend in newly reported HDV cases over the timeframes analyzed (Fig 2B). Significant MK trends were not observed in 7 (41%) HDV datasets. A significant difference in the frequency of increasing and decreasing HDV and HBV trends was detected with more HDV datasets increasing across the time series analyzed than HBV (Fisher exact, p<0.05). Divergent trends were observed in 4 (24%) countries with significant decreasing trends in HBV and increasing in HDV. Differences between HDV and HBV MK trends were positive in 14/17 datasets with HDV having a more positive MK trend compared to MK trend for HBV (Fig 2C). Only 3 datasets (United States, Brazil, Thailand) (Fig 2C), had higher HBV MK trend than HDV MK trend.

### Structural breaks in aggregated HDV incidence occurred in 2002, 2012, and 2017

**Fig 3:**
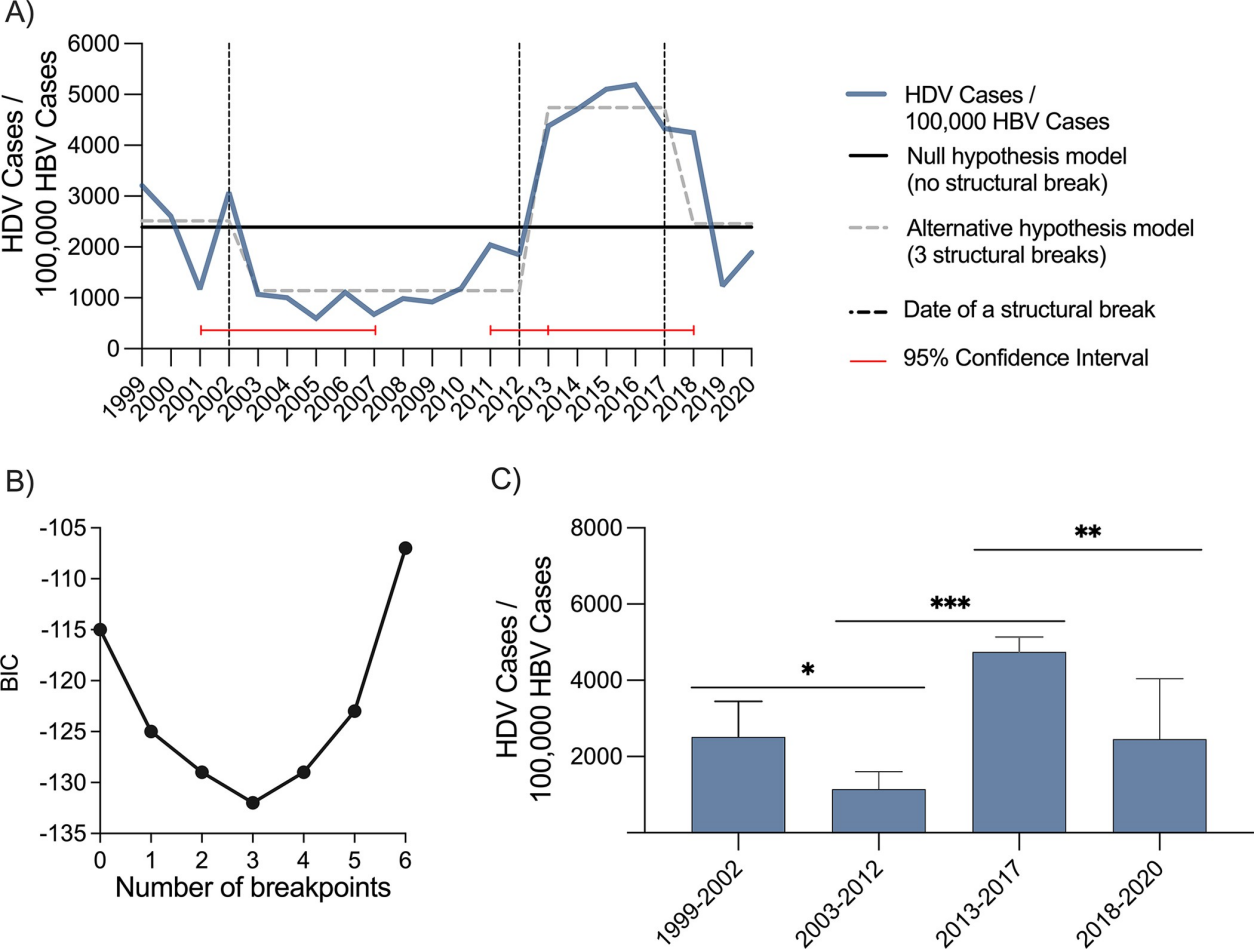
Structural break analysis identified three structural breaks at 2002, 2012 and 2017 in the aggregated yearly incidence of HDV/HBV_100,000_ cases. **A)** The presence of three structural breaks in average HDV/HBV_100,000_ cases occur in 2002, 2012 and 2017. Fitting the null hypothesis model (no structural break) and alternative hypothesis model (3 structural breaks) to the data indicate clear structural shifts at those times. **B)** BIC analysis identified 3 structural breaks in the aggregated HDV/HBV_100,000_ cases time series. **C)** HDV/HBV_100,000_ cases compared for 1999–2002, 2003–2012, 2013–2017 and 2018–2020. *p ≤ 0.05, **p ≤ 0.01, ***p ≤ 0.001.

Identification of a divergence in HDV and HBV incidence trends supported analysis of potential breakpoints in the HDV/HBV timeseries. Breakpoint analysis was performed to identify the potential presence and timeline of structural breaks in HDV incidence relative to reported HBV. An autoregressive integrated moving average (ARIMA) was calculated for the aggregated HDV/HBV time series. This HDV/HBV time series was fitted using a constant model with no structural break (null hypothesis model) and compared against a model with 1, 2, or more structural breaks (alternative hypothesis model) (Fig 3A). The Bayesian Information Criterion (BIC) was used to identify the presence and number of potential breaks in the time series. This analysis found that a model with 3 breakpoints represented the lowest BIC value and therefore was the best fitting model (Fig 3B).

Using this breakpoint analysis, the break dates in the aggregate HDV incidence data were identified as 2002, 2012, and 2017 with accompanying 95% confidence intervals (Fig 3A). As detailed in Fig 3C, a significant increase in HDV/HBV_100,000_ was observed between 2013–2017. Based on these breakpoints, the average yearly incidence of HDV significantly decreased between the periods of 1999–2002 (2,515 HDV/HBV_100,000_) and 2003–2012 (1,144 HDV/HBV_100,000_; Fold Change (FC): -2.2; p = 0.03), increased between the periods of 2003–2012 (1,144 HDV/HBV_100,000_) and 2013–2017 (4,741 HDV/HBV_100,000_; FC: 4.14; p<0.0001) and decreased again between the periods of 2013–2017 (4,741 HDV/HBV_100,000_) and 2018–2020 (2,458 HDV/HBV_100,000_; FC: -1.93; p = 0.003) (Fig 3C). HDV incidence for each country based on the identified breakpoints is detailed in S3 Fig.

### Identified correlations and clusters in HDV incidence in the international HDV datasets

**Fig 4:**
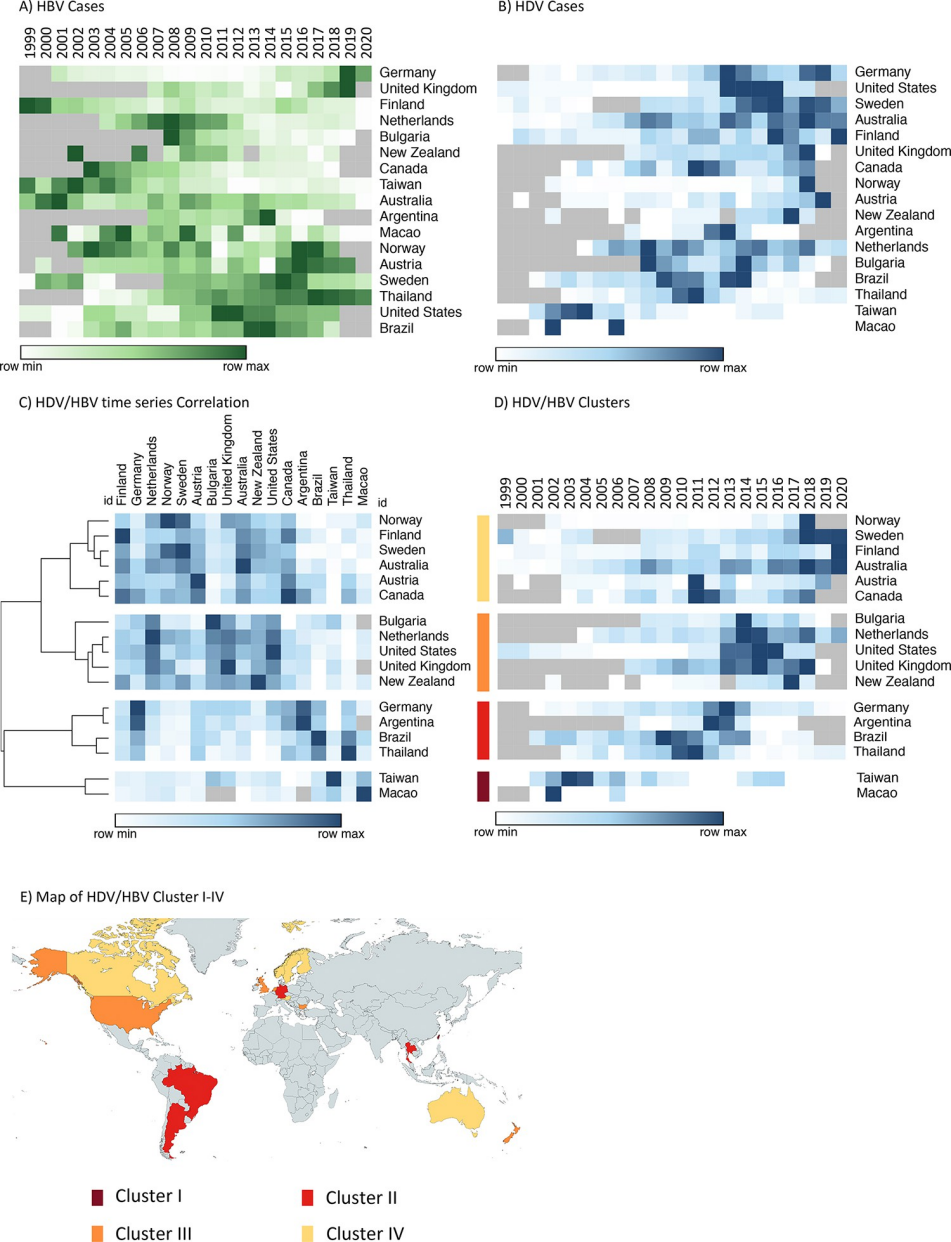
Hierarchal clustering of the HDV and HBV datasets identified distinct temporal clusters. **A)** Hierarchal clustering HBV cases and **B)** similar clustering order for HDV show a divergence in HDV and HBV yearly incidence for a subset of countries. **C)** Hierarchal clustering of the pairwise correlations of HDB/HBV among the 17 countries or region datasets analyzed identified four distinct clusters: Cluster I (Macao, Taiwan), Cluster II (Argentina, Brazil, Germany, Thailand), Cluster III (Bulgaria, Netherlands, New Zealand, United Kingdom, United States) and Cluster IV (Australia, Austria, Canada, Finland, Norway, Sweden). **D)** HDV/HBV yearly incidence within clusters identified a temporal trend in HDV/HBV incidence ranging from cluster I-IV. **E)** Map of HDV/HBV clusters show country locations for each cluster spans multiple continents, except Cluster I. Republished from https://www.mapchart.net/ under a CC BY license, with permission from MapChart original copyright 2022.

To further characterize the international incidence of HDV over time, the 17 HDV incidence datasets were evaluated for temporal pairwise correlations (Fig 4). Hierarchal cluster analysis was performed on HBV cases over the time-series analyzed (Fig 4A). Similar country alignment for HDV (Fig 4B) identified divergence from the HBV time series with a subset of countries having lower reported HBV cases and increased reported HDV cases.

In Fig 4C, hierarchal cluster analysis was performed on pairwise correlations of the 17 datasets and identified four distinct clusters, including Cluster I (Taiwan and Macao), Cluster II (Argentina, Brazil, Germany, and Thailand), Cluster III (Bulgaria, Netherlands, New Zealand, United Kingdom, and United States), and Cluster IV (Australia, Austria, Canada, Finland, Norway, Sweden). The heatmap of the maximal HDV/HBV yearly incidence for each individual country dataset identified 4 waves of increased HDV/HBV_100,000_ cases starting with Cluster I in the early 2000s to Cluster IV in the later 2010s (Fig 4D). Fig 4E details Cluster I-IV localization on the world map with clusters spanning multiple continents. Additional analyses were performed based on these identified temporal clusters.

### Structural breaks in HDV cluster analyses identified 4 waves of increased HDV incidence

**Fig 5:**
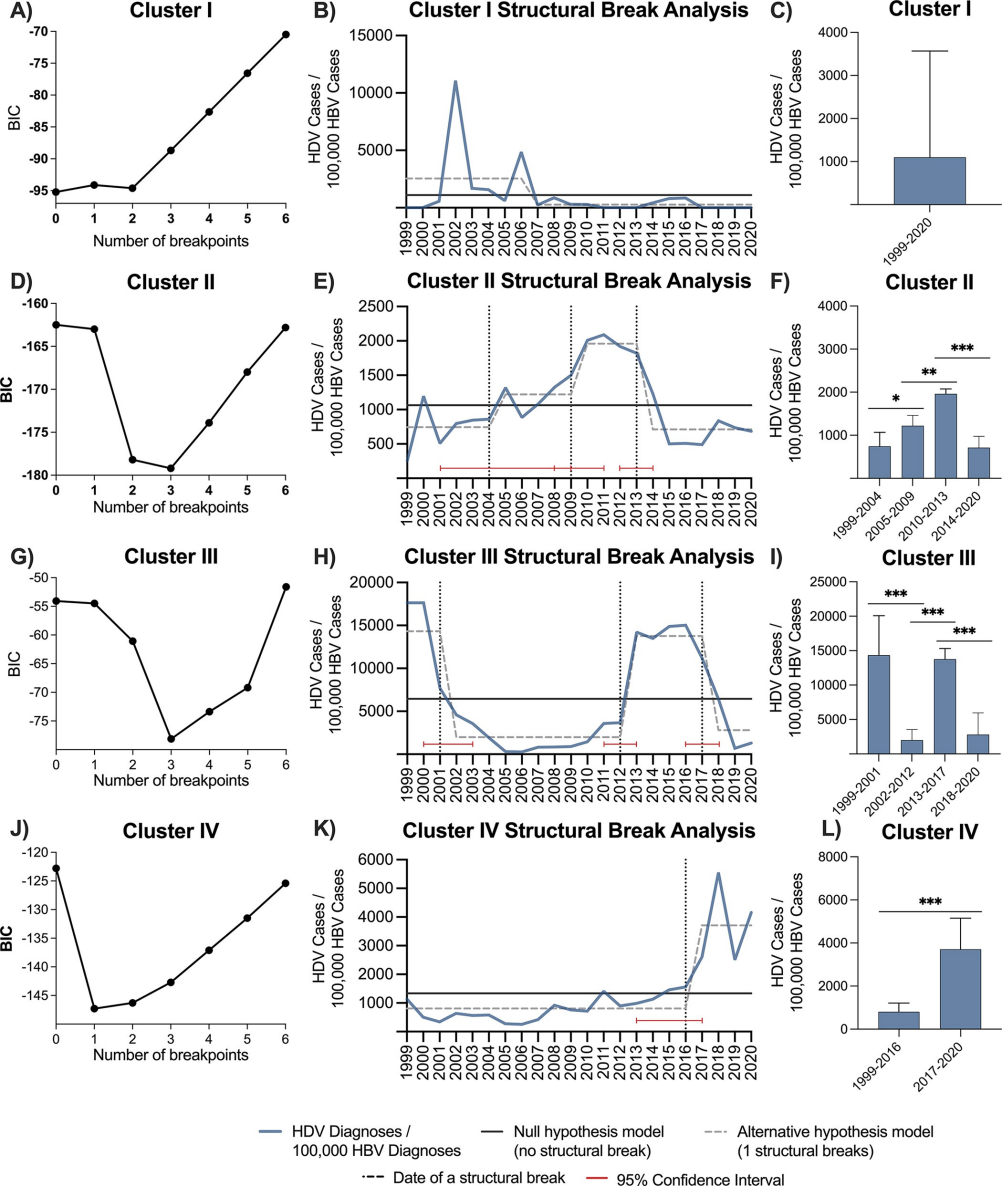
Structural break analysis of four clusters identified. **A, D, G, J)** BIC analysis identified number of breaks within Cluster I-IV. The lowest value on the BIC graph represents the number of breaks expected in each dataset analyzed. **B, E, H, K)** Timeline of HDV/HBV_100,000_ cases for Cluster I-IV. **C, F, I, L)** The average HDV/HBV_100,000_ cases for each of the breakpoints identified for Cluster I-IV. Cluster I had no significant structural breaks. Significant increases were noted in 2010–2013 for Cluster II, 1999–2001 and 2013–2017 for Cluster III and 2017–2020 for Cluster IV. Statistical analysis was performed for adjacent time series identified using standard multi-comparison t-test. *p ≤ 0.05, **p ≤ 0.01, ***p ≤ 0.001.

Breakpoint analysis was performed on each temporal cluster to determine the optimal number of breaks to use for developing the best-fitting models (Fig 5A, 5D, 5G and 5J). From these best-fitting models, the timelines of the structural breaks were identified (Fig 5B, 5E, 5H and 5K) and the average HDV/HBV_100,000_ cases for each breakpoint identified (Fig 5C, 5F, 5I and 5J). In Cluster I (Macao and Taiwan), no significant breakpoints were identified, but the maximal HDV/HBV_100,000_ was observed from 2002–2004 (Fig 5A–5C, S3 Table, S4A Fig).

In Cluster II (Argentina, Brazil, Germany, and Thailand), significant breakpoints were identified after 2004, 2009, and 2013 with the maximal HDV/HBV_100,000_ incidence occurring in 2010–2013 (1,959, 95% CI 1,775–2,143 HDV/HBV_100,000_) (Fig 5D–5F, S3 Table, S4B Fig).

In Cluster III (Bulgaria, Netherlands, New Zealand, United Kingdom, and United States), significant breakpoints were identified after 2001, 2012, and 2017, with the maximal HDV/HBV_100,000_ incidences occurring in 1999–2001 (14,329, 95% CI 51–28,606 HDV/HBV_100,000_), and 2013–2017 (13,763, 95% CI 11,825–15,701 HDV/HBV_100,000_) (Fig 5G–5I, S3 Table, S4C Fig).

In Cluster IV (Australia, Austria, Canada, Finland, Norway, and Sweden), a single significant breakpoint was identified after 2016, with the maximal HDV/HBV_100,000_ incidence occurring in 2017–2020 (1,222, 95% CI 924–1,520 HDV/HBV_100,000_) (Fig 5J–5L, S3 Table, S4D Fig).

## Discussion

Hepatitis Delta Virus (HDV) is currently defined as a rare infectious disease in many countries around the world [3, 31–33]. Due to this perceived low frequency of transmission, the tracking of HDV cases is attenuated by limited HDV testing of HBV-positive patient populations [6, 34] and the lack of active surveillance of HDV. This study provides an essential resource to enable tracking of HDV epidemiology through analysis of publicly accessible and actively updated infectious disease datasets. To date, 17 publicly accessible infectious disease datasets containing yearly incidence of newly reported HDV and HBV cases have been identified and analyzed in this study (S1 Table). Analyses of the aggregated and temporal clusters of the international HDV and HBV datasets revealed multiple recent and significant structural breaks in the timeline of newly reported HDV cases relative to HBV.

### Similar trends in HDV incidence

The observed increases of newly reported HDV cases share a similar pattern with recent publications on HDV epidemiology. In France, a study by Servant-Delmas et al. was conducted using viral data (1997–2011) from blood donors in the National Epidemiological Donors database. The incidence of HDV in HBV-positive blood donors transitioned from 1.1% in 1997–2005, 4.2% in 2006, and 6.5% in 2010, before declining to 0.85% in 2011 [35]. This transition occurred during a time period of steady decline in HBV diagnoses in blood donations. In Iran, Sayad et al. evaluated HDV seroprevalence in HBV-positive patients and noted fluctuations from 2.15% in 2004 to 1.28% in 2010, then back up to 3.47% in 2014, before declining again to 0.89% in 2016 [36]. A recent publication evaluating HBV and HDV epidemiology from Yakutia, Russia reported increasing HDV population in connection with a stable HBV prevalence [37]. Similar patterns of HDV incidence were also reported in Cameroon, Austria, and United States [38–41]. These reported shifts in HDV incidence mirror some of the structural breaks observed in our analyses. Together, these internationally shared structural breaks in the incidence of HDV warrant enhanced surveillance to monitor for emerging uncharacteristic transmission patterns of HDV.

### Potential factors impacting HDV epidemiology

Multiple factors may influence the perceived increase in the yearly incidence of HDV/HBV cases. A majority of publicly available infectious disease datasets do not report the type of tests used, the number of tests performed or whether duplicate diagnoses are present in the HDV and HBV infectious disease datasets. Additionally, clinical studies evaluating new therapies for HDV treatment are underway and may contribute to increased testing due to raised community awareness [42–44]. Differences in the testing guidelines, reporting requirements, community awareness of HDV and frequency of HDV testing in HBV patients could significantly impact the yearly reported HDV cases.

The differences in HDV testing guidelines and reporting may significantly impact the reported number of cases each year. An example of this is noted within North America. Canada (British Columbia) reported 0.26% HDV/HBV diagnoses and NHANES (United States) reported 20% HDV/HBV diagnoses (Table 1). This significant divergence in reported prevalence of HDV may be attributed to differences in testing approach and/or guidelines. NHANES tests a sampling in the United States population for HBV whether or not subjects have symptoms of hepatitis and test all HBV surface antigen (HBSAg)-positive patients for the presence of HDV antibodies [45, 46]. This is in contrast to standard testing protocols that rely on detection of HBV to trigger HDV testing and, only then, a limited number of HBV patients are tested for evidence of an HDV co-infection. In the United States, multiple studies have reported that less than 20% of HBV patients are tested for HDV[6, 34, 47–49]. Therefore, national testing guidelines, local reporting requirements and compliance in testing HBV patients for HDV may significantly impact the reported cases of HBV and HDV. Increased active surveillance and reporting of HDV and HBV is justified for further defining the etiology of the observed structural breaks in the international incidence of HDV.

### Divergent trends in HDV and HBV epidemiology

Hepatitis Delta Virus and HBV transmission are predominantly associated with parenteral, vertical, and sexual exposure [1, 50, 51]. Therefore, given these established routes of HDV transmission with HBV, a similar trend would be anticipated between reported diagnoses of both viruses. As noted in Fig 2, 24% of countries with significantly decreasing HBV incidence had significantly increasing HDV incidence. Overall, 76% of the datasets analyzed had higher Mann-Kendall trends for HDV relative to HBV. The underlying cause(s) of these opposing trends in HDV and HBV epidemiology are undefined and was further investigated through cluster analysis.

In our cluster analysis, correlating trends in HDV incidence were identified in some geographically dispersed and seemingly unrelated countries. Due to the nature of HDV and HBV transmission, a shared pattern of increased HDV diagnoses spanning multiple continents was not anticipated. Multiple factors may trigger similar trends in geographically dispersed countries, including concerted changes in the policy of HDV testing guidelines and reporting requirements, international trends in intravenous drug use (IVDU) or other risk factors associated with HDV transmission [52], or shared alternative routes of HDV transmission (ex. alternative HDV reservoirs). Additional studies are now required to identify the underlying etiology and connection between these international clusters.

### Increasing HDV testing may identify increased HDV prevalence

Increased reflex testing for HDV in HBV-positive patients may identify higher prevalence of HDV. The United States’ NHANES HDV and HBV datasets are acquired biennially within the general population through cross-sectional study approach, and HBV testing occurs independently of reported indicators of hepatitis [46]. This is different than the passive reporting of HDV positive tests reported by government agencies. All NHANES subjects that test positive for both HBV core antibodies (HBc-Ab) and surface antigens (HBsAg) are then tested for HDV antibodies [45]. The dataset from the United States, utilizing the CDC’s NHANES dataset, detected HDV in >50% of HBV-positive subjects tested (2013–2016). This may indicate widespread occurrence of HDV in asymptomatic HBV carriers and in patients that may not fit recommended testing guidelines [52, 53]. The significant increase in newly reported HDV cases among HBV-positive subjects suggests that the current HDV testing guidelines need to be reassessed. This is further demonstrated with the new European Association for the Study of the Liver (EASL) and the Asian Pacific Association for the Study of the Liver (APASL) testing guidelines recommending that all HBV patients be tested for HDV [54, 55]. Palom et al recentl reported the detection of 5 times more HDV cases under EASL’s new HDV testing guidelines [56]. Mandating automatic reflex testing for HDV in HBV-positive populations may render a significant increase in the prevalence of HDV. Currently, chronic HBV infections are estimated to impact >1.5 million people in the United States population and >200 million worldwide [57, 58].

Our knowledge of Hepatitis Delta Virus is rapidly evolving. Multiple recent publications have noted the capacity of HDV and HDV-like sequences to package into non-HBV particles [59, 60], and at least eight new HDV-like sequences have been detected in diverse animal species, including birds, bats, deer, rodents, snakes, newts, toads, and termites [10–14, 17]. Additionally, HDV has been detected in human salivary gland tissue in the absence of an evident current or past HBV co-infection [8]. Together, these recently reported deviations in the established HDV virology further demonstrate the need for enhanced active surveillance of HDV to track the emergence of new HDV variants and/or alternative patterns of transmission. The detected changes in HDV epidemiology on an international scale warrant increased active surveillance and sharing of this de-identified data through publicly accessible databases.

## Conclusion

This study has identified several publicly accessible, actively updating international infectious diseases datasets that support the ongoing analysis of HDV epidemiology. The datasets utilized ranged between 1999–2020 and spanned 5 continents. Our analysis identified divergent trends in HDV and HBV transmission, structural breaks in the aggregated HDV time series, and four temporal clusters of HDV cases. Increased surveillance of HDV is warranted to further define the etiology of the recent breakpoints in HDV incidence spanning multiple continents.

## Supporting information

S1 Table Publicly accessible datasets used in analysis of HDV and HBV diagnoses. (PDF)

S2 Table Mann-Kendall Trends in time series for newly reported HDV and HBV cases. Mann-Kendall analyses were performed to identify trends in the incidence of HDV and HBV over the time series analyzed. Kental Tau, Mann-Kendall (MK) analysis, p-value and Sen Slope reported for each country or region HDV and HBV dataset. Graphical view of MK analyses are depicted in Fig 2 and S2 Fig.
(PDF)

S3 Table Cluster HDV analysis statistics. (PDF)

S1 Fig Yearly reported cases for HDV and HBV. Publicly accessible infectious disease datasets containing yearly incidence of HDV and HBV for 17 countries or region spanning 5 continents were utilized in analyses. Data ranges between 1999–2020. Details of years reported by each country or region is detailed in Table 1.
(PDF)

S2 Fig Significant divergence in HDV and HBV time series trends identified in Kendall Tau and Sen Slope analyses. **A-C)** Kendall Tau analysis for HBV, HDV and the difference identified significant trends in HBV and HDV timeseries. 58% of HBV datasets presented with a decreasing trend over the time series evaluated. In contrast, 76% of the HDV datasets presented with increasing trends over the time series evaluated. 82% (14/17) of the HDV and HBV datasets showed a divergent trend between HDV and HBV or a slower decline in HDV relative to changes in HBV. Three datasets, United States (NHANES), Brazil and Thailand, presented with increasing HBV trends relative to HDV. **D-E)** Sen Slope measures the degree of change in the time series trends. This analysis identified an overall trend of increasing incidence of HDV over the times series evaluated.
(PDF)

S3 Fig HDV cases/100,000 HBV cases for each country based on structural breaks identified for aggregated dataset. Structural breaks were identified in 2002, 2013, and 2017. Comparison of the identified timeframes 1999–2002, 2003–2012, 2013–2017 and 2018–2020 for each country or region are depicted. Grouping of countries or region are based of continent location and/or scale of HDV cases/100,000 HBV cases. Comparison of HDV/HBV incidence for identified breakpoints in the aggregated data, Country level analyses for A) Asia and Australia, B) Europe, C) Americas, and D) United States. *p ≤ 0.05, **p ≤ 0.01, ***p ≤ 0.001.
(PDF)

S4 Fig Country or region level newly reported HDV cases per 100,000 HBV cases for identified Clusters II, III and IV. A) No structural breaks and differences were identified for cluster I. B) Comparisons of years 1999–2001, 2002–2012, 2013–2017 and 2018–2020 in Cluster II. C) Comparison of 1999–2004, 2005–2009, 2010–2013 and 2014–2020 in Cluster III. D) Comparison of 1999–2016 and 2017–2020 in Cluster IV. E-H) Country-level comparisons of HDV incidence for identified clusters based on respective breakpoints. *p ≤ 0.05, **p ≤ 0.01, ***p ≤ 0.001.
(PDF)

## List of abbreviations

CDC: Center for Disease Control
NHANES: National Health and Nutritional Examination Survey
BIC: Bayesian Information Criterion
HDV: Hepatitis Delta Virus
HBV: Hepatitis B Virus
