# Supplementary material for: A changing landscape: Tracking and analysis of the international HDV epidemiology 1999–2020
Source: PLOS Glob Public Health. 2023 Apr 25;3(4):e0000790. doi: 10.1371/journal.pgph.0000790 (PMC10129014; doi:10.1371/journal.pgph.0000790)
Supplement: S1 Table — (PDF) [file pgph.0000790.s001.pdf]

S1 Table. Publicly accessible datasets used in analysis of newly reported HDV and HBV diagnoses.

Country	Website(s)
Argentina	Argentina Ministry of Health (http://www.msal.gob.ar/index.php/home/boletin-integrado-de-vigilancia)
	Argentina Ministry of Health (https://www.argentina.gob.ar/salud/epidemiologia/publicaciones)
	Argentina Ministry of Health (http://www.msal.gob.ar/images/stories/bes/graficos/0000001592cnt-2019-10_boletin-hepatitis.pdf)
Australia	Australian Government Department of Health (http://www9.health.gov.au/cda/source/cda-index.cfm)
Austria	Austrian Federal Ministry of Social Affairs, Health, Care and Consumer Protection (https://www.sozialministerium.at/Themen/Gesundheit/Uebertragbare-Krankheiten/Statistiken-und-Fallzahlen.html)
	Statistik Austria (https://statistik.at/web_de/nomenu/suchergebnisse/index.html?searchQuery=Jahrbuch%20der%20Gesundheitsstatistik)
	Statistik Austria (https://www.statistik.at/web_de/services/publikationen/4/index.html)
Brazil †	Brazil Health Information (TABNET) (SINANWIN) (http://tabnet.datasus.gov.br/cgi/deftohtm.exe?sinanwin/cnv/hepabr.def)
	Brazil Health Information (TABNET) (SINANNET) (http://tabnet.datasus.gov.br/cgi/deftohtm.exe?sinannet/cnv/hepabr.def)
Bulgaria	Bulgaria National Center of Infectious and Parasitic Diseases (https://ncipd.org/index.php?option=com_k2&view=item&layout=item&id=84&Itemid=1337)
Canada	BC Centre for Disease Control (http://www.bccdc.ca/health-professionals/data-reports/reportable-diseases-data-dashboard)
Finland	Finland Department of Health and Welfare (https://www.thl.fi/ttr/gen/rpt/tilastot.html)
Germany	Infectious Disease Epidemiology Annual Report – Robert Koch Institute (http://www.rki.de/DE/Content/Infekt/Jahrbuch/jahrbuch_node.html)
	SurvStat – Robert Koch Institute (https://survstat.rki.de/Content/Query/Create.aspx)
Macao	Macao Special Administrative Region Government Health Services (http://www.ssm.gov.mo/Portal/portal.aspx?lang=pt)
Netherlands	Netherlands National Institute of Public Health and Environment (https://www.rivm.nl/infectieziekten-bulletin)
New Zealand	New Zealand Ministry of Health – Public Health Surveillance (https://surv.esr.cri.nz/PDF_surveillance/MthSurvRpt)
	New Zealand Ministry of Health – Annual Surveillance Summary (https://surv.esr.cri.nz/surveillance/annual_surveillance.php)
Norway	Norway National Institute of Public Health (https://www.fhi.no/nettpub/smittevernveilederen/sykdommer-a-a/hepatitt-d/)
	Norway National Institute of Public Health – Laboratory Diagnostics (http://lab.fhi.no/Default.aspx)
Sweden	Swedish Public Health Agency – Hepatitis D (https://www.folkhalsomyndigheten.se/folkhalsorapportering-statistik/statistik-a-o/sjukdomsstatistik/hepatit-d)
	Swedish Public Health Agency – Hepatitis D (by county) (https://www.folkhalsomyndigheten.se/folkhalsorapportering-statistik/statistik-a-o/sjukdomsstatistik/hepatit-d/?t=county)
	Swedish Public Health Agency – Hepatitis B (https://www.folkhalsomyndigheten.se/folkhalsorapportering-statistik/statistik-a-o/sjukdomsstatistik/hepatit-b/?p=5671)
	Swedish Public Health Agency – Hepatitis B (by county) (https://www.folkhalsomyndigheten.se/folkhalsorapportering-statistik/statistik-a-o/sjukdomsstatistik/hepatit-b/?t=county)
Taiwan	Taiwan Centers for Disease Control (http://www.cdc.gov.tw/english/list.aspx?treeid=00ed75d6c887bb27&nowtreeid=c4e6b232ee7b2894)
	Taiwan Center for Disease Control – Hepatitis D (https://data.cdc.gov.tw/en/dataset/aagstable-acute-hepatitis-d)
	Taiwan Center for Disease Control – Hepatitis B (https://data.cdc.gov.tw/en/dataset/aagstable-acute-hepatitis-b)
	Taiwan Center for Disease Control – Disease Lookup (http://nidss.cdc.gov.tw/en/SingleDisease.aspx?dc=1&dt=3&disease=0703)
Thailand	Thailand Bureau of Epidemiology (http://www.boe.moph.go.th/boedb/surdata/disease.php?dcontent=old&ds=69)
United Kingdom	UK Health Protection Agency – Health Protection Report Archives (http://webarchive.nationalarchives.gov.uk/20140714090232/http://www.hpa.org.uk/hpr/archives/2013/news.htm)
	UK Government Publications (https://www.gov.uk/government/publications)
	UK Health Protection Report (https://www.gov.uk/government/publications/health-protection-report-volume-13-2019)
	UK Notification of Infectious Diseases (https://www.gov.uk/government/collections/notifications-of-infectious-diseases-noids#reports)
	UK Blood Born Viruses (https://www.gov.uk/government/publications/sentinel-surveillance-of-blood-borne-virus-testing-in-england-2016)
United States	United States Centers for Disease Control – NHANES (https://www.cdc.gov/nchs/nhanes/index.htm)

† For Brazil, the first link contains years 2001-2006 and the second link contains years 2007-present
