# Supplementary material for: A changing landscape: Tracking and analysis of the international HDV epidemiology 1999–2020
Source: PLOS Glob Public Health. 2023 Apr 25;3(4):e0000790. doi: 10.1371/journal.pgph.0000790 (PMC10129014; doi:10.1371/journal.pgph.0000790)
Supplement: S2 Table — Mann-Kendall analyses were performed to identify trends in the incidence of HDV and HBV over the time series analyzed. Kental Tau, Mann-Kendall (MK) analysis, p-value and Sen Slope reported for each country or region HDV and HBV dataset. Graphical view of MK analyses are depicted in Fig 2 and S2 Fig. (PDF) [file pgph.0000790.s002.pdf]

S2 Table. Mann-Kendall Trends in time series for reported HDV and HBV cases. Mann-Kendall analyses were performed to identify trends in the incidence of HDV and HBV over the time series analyzed. Kendall Tau, Mann-Kendall (MK) analysis, p-value and Sen Slope are reported for each country or region HDV and HBV dataset. Graphical view of MK analyses are depicted in Fig 2 and S2 Fig.

	Countries	Years in Dataset	HDV Kendall Tau	HDV Mann-Kendall	p-value	HDV Sen Slope	HBV Kendall Tau	HBV Mann-Kendall	p-value	HBV Sen Slope
Europe	Austria	1999-2019	0.744	97	<0.001	0.556	-0.359	92	0.021	-55.909
	Bulgaria	2008-2019	-0.526	-32	0.028	-0.429	-0.909	-60	<0.001	-25.625
	Finland	1999-2020	0.493	106	0.002	0.182	-0.587	-135	<0.001	-10
	Germany	2001-2020	0.677	128	<0.001	1.785	0.179	34	n.s.	78.077
	Netherlands	2004-2020	0.008	1	n.s.	0	-0.441	-60	0.015	-55.045
	Norway	2002-2018	0.229	30	n.s.	0.675	-0.147	-20	n.s.	-11.464
	Sweden	1999-2004, 2008-2020	0.629	107	<0.001	3	0.088	15	n.s.	7.667
	United Kingdom	2007-2019	0.468	36	0.032	4.292	0.051	4	n.s.	37.625
North America	United States	1999-2018	0.321	56	n.s.	0.303	0.497	92	0.003	1
	Canada	2003-2018	0.414	48	0.033	0.268	-0.845	-101	<0.001	-47.187
Australia	Australia	1999-2020	0.662	152	<0.001	2.571	-0.359	-83	0.021	-55.909
	New Zealand	2002, 2006, 2008-2018	0.691	42	0.004	0.333	-0.813	-52	<0.001	-2.429
South America	Argentina	2007-2017	-0.042	-2	n.s.	0	-0.345	-19	n.s.	-27.75
	Brazil	2001-2018	0.059	9	n.s.	1.714	0.438	67	0.012	297.25
Asia	Macao	2000-2020	-0.314	-26	n.s.	0	-0.43	-80	0.01	-0.655
	Taiwan	1999-2020	-0.392	-81	0.018	-0.2	-0.694	-160	<0.001	-13.5
	Thailand	1999-2020	0.052	8	n.s.	0.357	0.817	125	<0.001	273.133
