# Supplementary material for: A changing landscape: Tracking and analysis of the international HDV epidemiology 1999–2020
Source: PLOS Glob Public Health. 2023 Apr 25;3(4):e0000790. doi: 10.1371/journal.pgph.0000790 (PMC10129014; doi:10.1371/journal.pgph.0000790)
Supplement: S3 Table — (PDF) [file pgph.0000790.s003.pdf]

S3 Table. Cluster HDV analysis descriptive statistics.

Cluster II	Mean (95% CI) HDV/HBV$_{100,000}$	ANOVA
**1999-2004**	743.9 (404.4 to 1083)	F(3,18) = 23.77 p<0.0001
**2005-2009**	1222 (923.7 to 1520)	
**2010-2013**	1959 (1775 to 2143)	
**2014-2020**	712.7 (468 to 957.4)	

Comparisons	Fold Change	P-Value
**1999-2004 vs. 2005-2009**	1.64	0.0325
**1999-2004 vs. 2010-2013**	2.63	<0.0001
**1999-2004 vs. 2014-2020**	-1.04	0.9963
**2005-2009 vs. 2010-2013**	1.60	0.0026
**2005-2009 vs. 2014-2020**	-1.71	0.0171
**2010-2013 vs. 2014-2020**	-2.75	<0.0001

Cluster III	Mean (95% CI) HDV/HBV$_{100,000}$	ANOVA
**1999-2001**	14329 (51.48 to 28606)	F(3,18) = 35.56 p<0.0001
**2002-2012**	1992 (938 to 3045)	
**2013-2017**	13763 (11825 to 15701)	
**2018-2020**	2808 (-4999 to 10615)	

Comparisons	Fold Change	P-Value
**1999-2001 vs. 2002-2012**	-7.19	<0.0001
**1999-2001 vs. 2013-2017**	-1.04	0.9903
**1999-2001 vs. 2018-2020**	-5.10	0.0002
**2002-2012 vs. 2013-2017**	6.91	<0.0001
**2002-2012 vs. 2018-2020**	1.41	0.9614
**2013-2017 vs. 2018-2020**	-4.90	<0.0001

Cluster IV	Mean (95% CI) HDV/HBV$_{100,000}$	F-Statistic
**1999-2016**	743.9 (404.4 to 1083)	F(3,17) = 12.70 p=0.0003
**2017-2020**	1222 (923.7 to 1520)	

Comparisons	Fold Change	P-Value
**1999-2016 vs. 2017-2020**	4.59	<0.0001
