# Supplementary material for: A changing landscape: Tracking and analysis of the international HDV epidemiology 1999–2020
Source: PLOS Glob Public Health. 2023 Apr 25;3(4):e0000790. doi: 10.1371/journal.pgph.0000790 (PMC10129014; doi:10.1371/journal.pgph.0000790)
Supplement: S1 Fig — Publicly accessible infectious disease datasets containing yearly incidence of HDV and HBV for 17 countries or region spanning 5 continents were utilized in analyses. Data ranges between 1999–2020. Details of years reported by each country or region is detailed in Table 1. (PDF) [file pgph.0000790.s004.pdf]

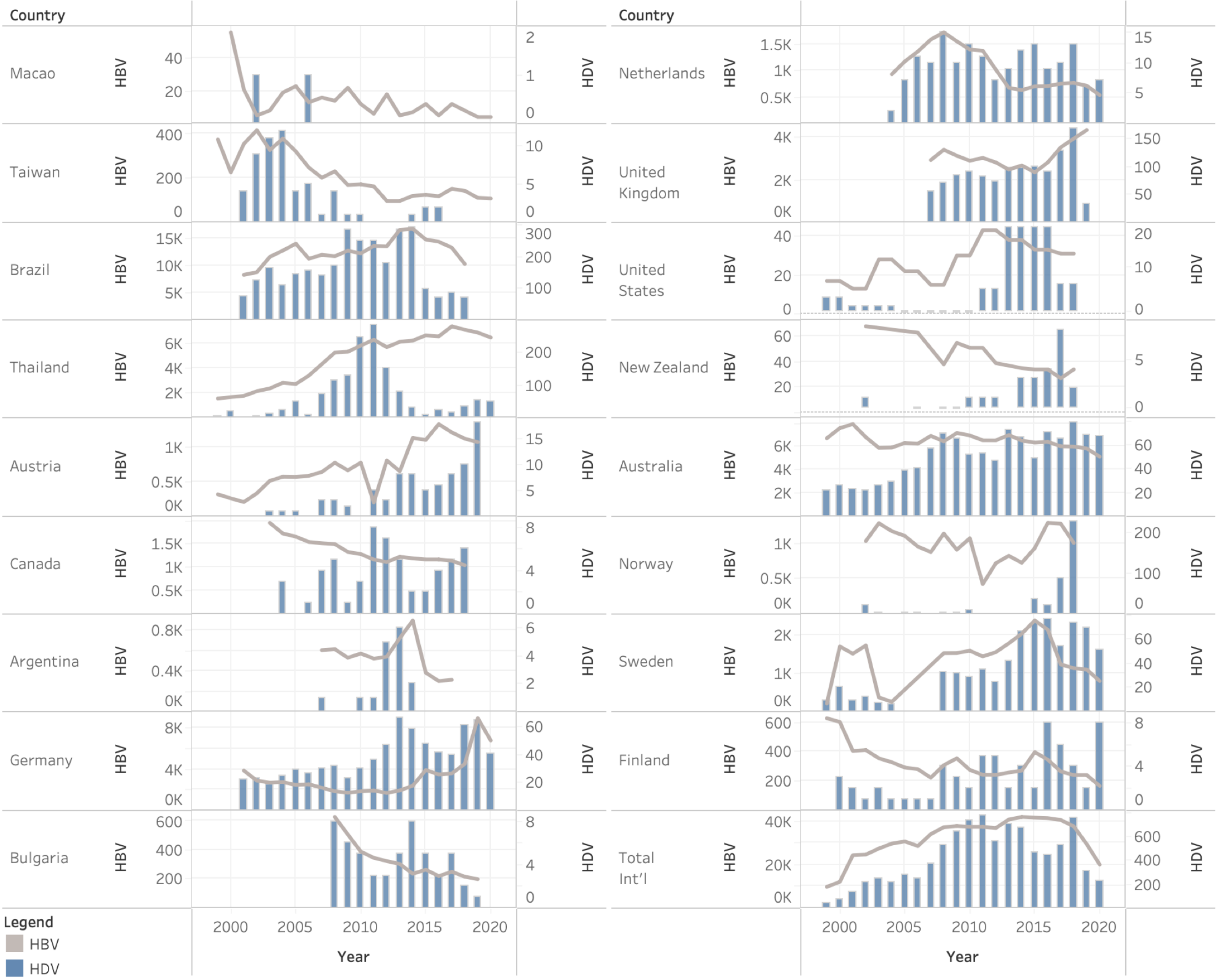

S1 Fig. Yearly newly reported HDV and HBV cases. Publicly accessible infectious disease datasets containing yearly incidence of HDV and HBV for 17 countries or region spanning 5 continents were utilized in analyses. Data ranges between 1999-2020. Details of years reported by each country or region is detailed in Table 1.
