# Supplementary material for: A changing landscape: Tracking and analysis of the international HDV epidemiology 1999–2020
Source: PLOS Glob Public Health. 2023 Apr 25;3(4):e0000790. doi: 10.1371/journal.pgph.0000790 (PMC10129014; doi:10.1371/journal.pgph.0000790)
Supplement: S2 Fig — A-C) Kendall Tau analysis for HBV, HDV and the difference identified significant trends in HBV and HDV timeseries. 58% of HBV datasets presented with a decreasing trend over the time series evaluated. In contrast, 76% of the HDV datasets presented with increasing trends over the time series evaluated. 82% (14/17) of the HDV and HBV datasets showed a divergent trend between HDV and HBV or a slower decline in HDV relative to changes in HBV. Three datasets, United States (NHANES), Brazil and Thailand, presented with increasing HBV trends relative to HDV. D-E) Sen Slope measures the degree of change in the time series trends. This analysis identified an overall trend of increasing incidence of HDV over the times series evaluated. (PDF) [file pgph.0000790.s005.pdf]

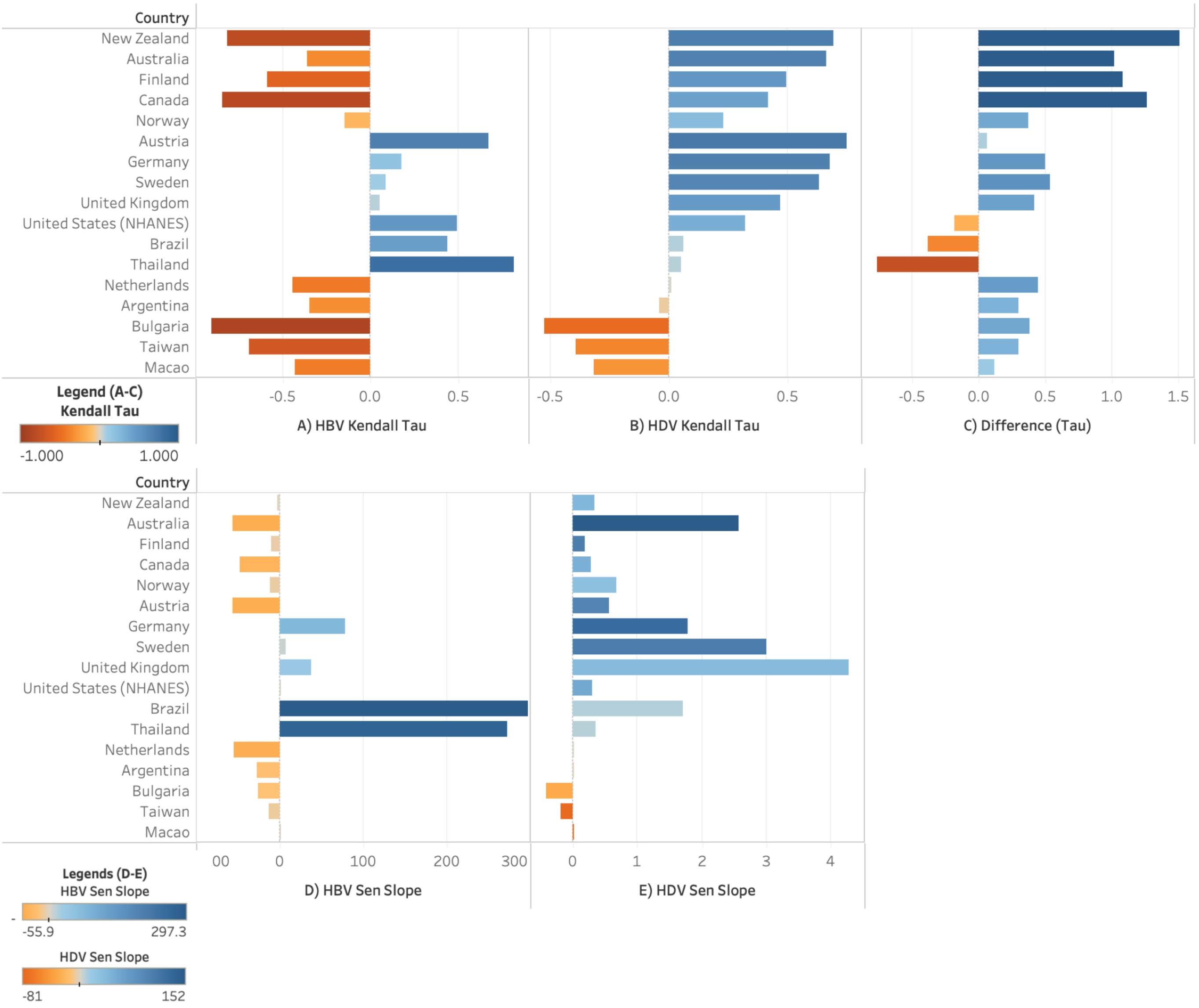

S2 Fig. Significant divergence in HDV and HBV time series trends identified in Kendall Tau and Sen Slope analyses. A-C) Kendall Tau analysis for HBV, HDV and the difference identified significant trends in HBV and HDV timeseries. 58% of HBV datasets presented with a decreasing trend over the time series evaluated. In contrast, 76% of the HDV datasets presented with increasing trends over the time series evaluated. 82% (14/17) of the HDV and HBV datasets showed a divergent trend between HDV and HBV or a slower decline in HDV relative to changes in HBV. Three datasets, United States (NHANES), Brazil and Thailand, presented with increasing HBV trends relative to HDV. **D-E)** Sen Slope measures the degree of change in the time series trends. This analysis identified an overall trend of increasing incidence of HDV over the times series evaluated.
