# Supplementary material for: A changing landscape: Tracking and analysis of the international HDV epidemiology 1999–2020
Source: PLOS Glob Public Health. 2023 Apr 25;3(4):e0000790. doi: 10.1371/journal.pgph.0000790 (PMC10129014; doi:10.1371/journal.pgph.0000790)
Supplement: S3 Fig — Structural breaks were identified in 2002, 2013, and 2017. Comparison of the identified timeframes 1999–2002, 2003–2012, 2013–2017 and 2018–2020 for each country or region are depicted. Grouping of countries or region are based of continent location and/or scale of HDV cases/100,000 HBV cases. Comparison of HDV/HBV incidence for identified breakpoints in the aggregated data, Country level analyses for A) Asia and Australia, B) Europe, C) Americas, and D) United States. *p ≤ 0.05, **p ≤ 0.01, ***p ≤ 0.001. (PDF) [file pgph.0000790.s006.pdf]

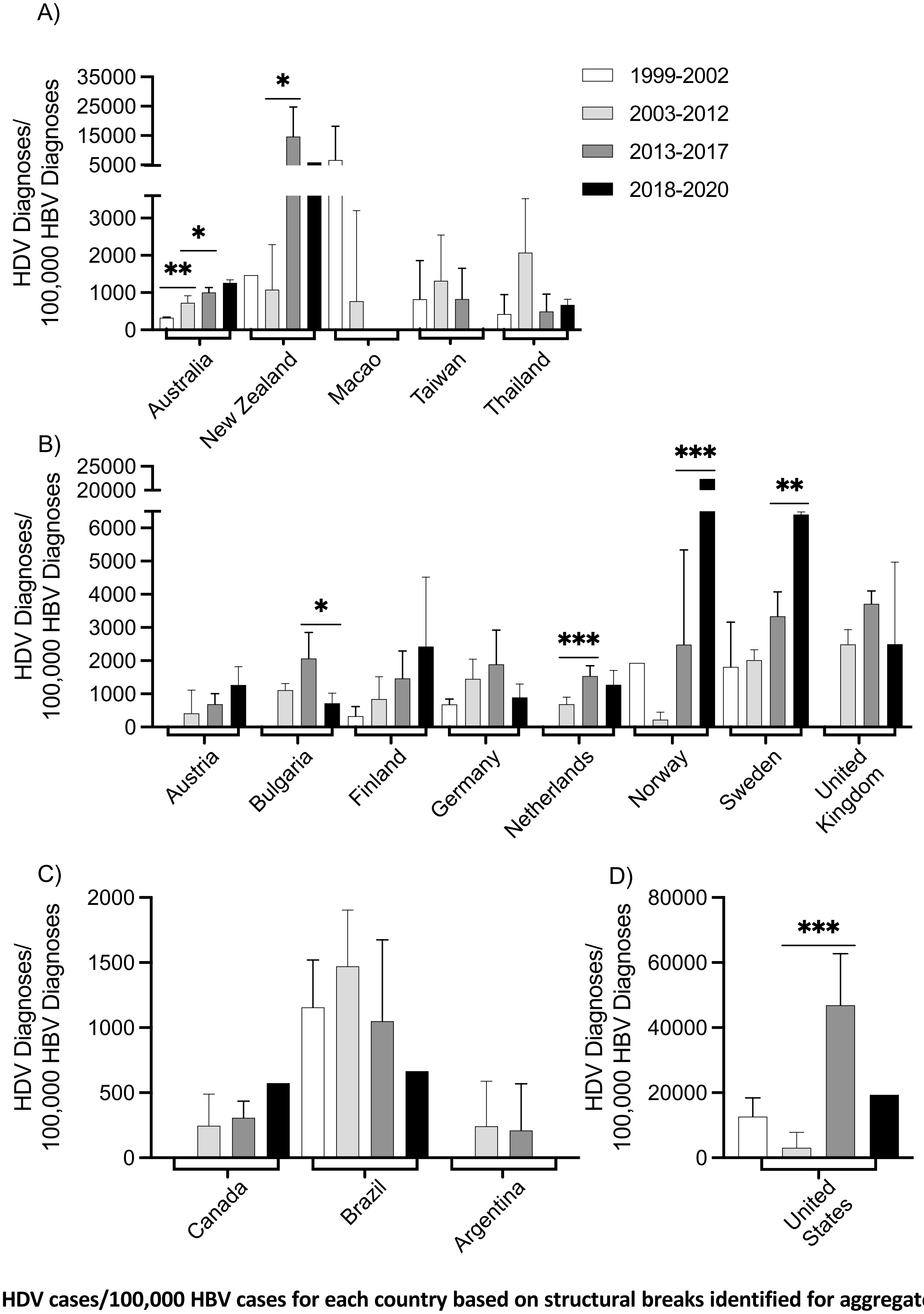

S3 Fig. HDV cases/100,000 HBV cases for each country based on structural breaks identified for aggregated dataset. Structural breaks were identified in 2002, 2013, and 2017. Comparison of the identified timeframes 1999-2002, 2003-2012, 2013-2017 and 2018-2020 for each country or region are depicted. Grouping of countries or region are based of continent location and/or scale of **HDV cases/100,000 HBV cases**. Comparison of HDV/HBV incidence for identified breakpoints in the aggregated data, Country level analyses for A) Asia and Australia, B) Europe, C) Americas, and D) United States. *p ≤ 0.05, **p ≤ 0.01, ***p ≤ 0.001
