# Supplementary material for: A changing landscape: Tracking and analysis of the international HDV epidemiology 1999–2020
Source: PLOS Glob Public Health. 2023 Apr 25;3(4):e0000790. doi: 10.1371/journal.pgph.0000790 (PMC10129014; doi:10.1371/journal.pgph.0000790)
Supplement: S4 Fig — A) No structural breaks and differences were identified for cluster I. B) Comparisons of years 1999–2001, 2002–2012, 2013–2017 and 2018–2020 in Cluster II. C) Comparison of 1999–2004, 2005–2009, 2010–2013 and 2014–2020 in Cluster III. D) Comparison of 1999–2016 and 2017–2020 in Cluster IV. E-H) Country-level comparisons of HDV incidence for identified clusters based on respective breakpoints. *p ≤ 0.05, **p ≤ 0.01, ***p ≤ 0.001. (PDF) [file pgph.0000790.s007.pdf]

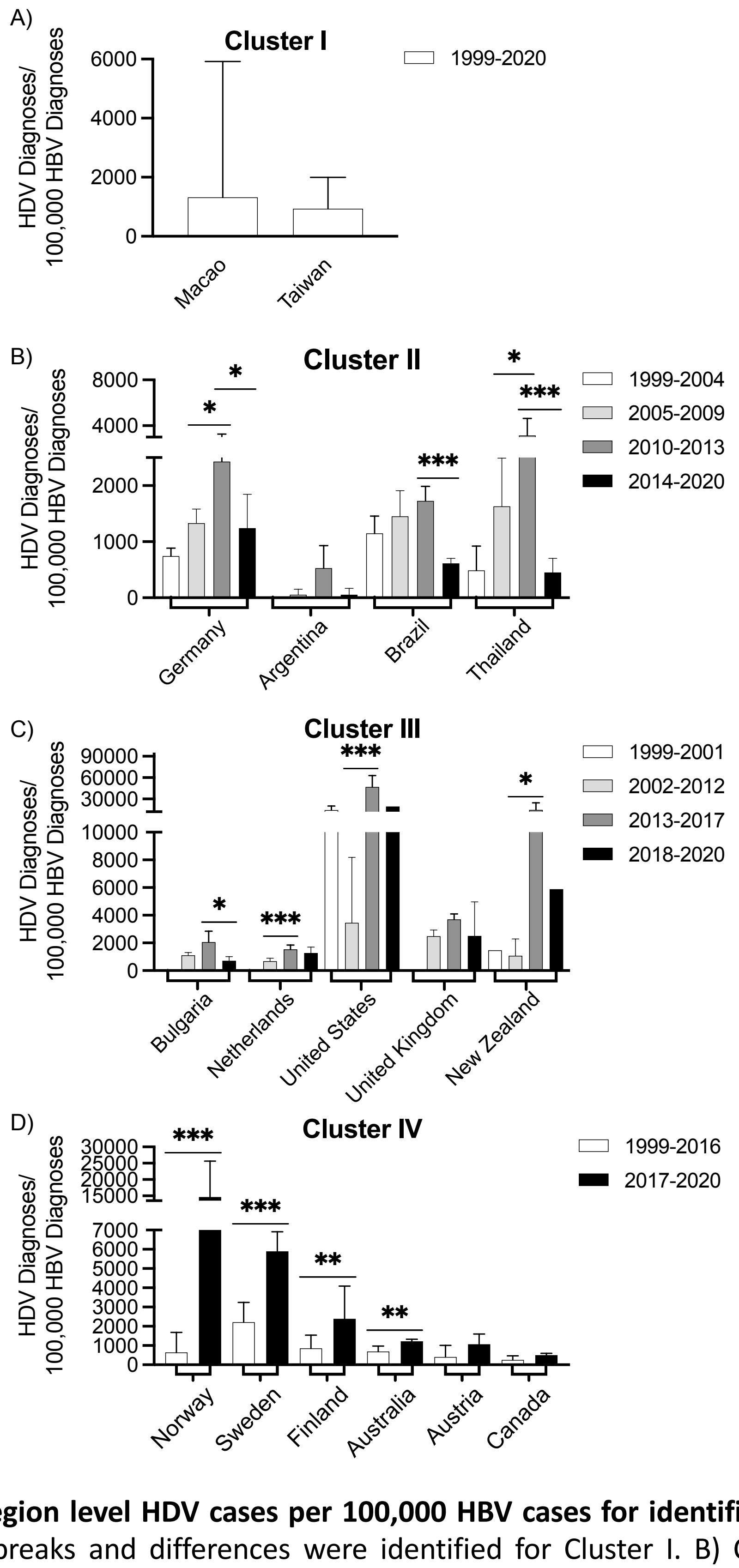

S4 Fig. Country or region level HDV cases per 100,000 HBV cases for identified Clusters II, III and IV. A) No structural breaks and differences were identified for Cluster I. B) Comparisons of years 1999-2001, 2002-2012, 2013-2017 and 2018-2020 in Cluster II. C) Comparison of 1999-2004, 2005-2009, 2010-2013 and 2014-2020 in Cluster III. D) Comparison of 1999-2016 and 2017-2020 in Cluster IV. *p ≤ 0.05, **p ≤ 0.01, ***p ≤ 0.001
